# Quality of life after open *versus* laparoscopic distal pancreatectomy: long-term results from a randomized clinical trial

**DOI:** 10.1093/bjsopen/zrad002

**Published:** 2023-03-09

**Authors:** Karin Johansen, Anna Lindhoff Larsson, Linda Lundgren, Thomas Gasslander, Claes Hjalmarsson, Per Sandström, Bergthor Björnsson

**Affiliations:** Department of Surgery in Linköping and Department of Biomedical and Clinical Sciences, Linköping University, Linköping, Sweden; Department of Surgery in Linköping and Department of Biomedical and Clinical Sciences, Linköping University, Linköping, Sweden; Department of Surgery in Linköping and Department of Biomedical and Clinical Sciences, Linköping University, Linköping, Sweden; Department of Surgery in Linköping and Department of Biomedical and Clinical Sciences, Linköping University, Linköping, Sweden; Department of Surgery, Hospital of Halland, Halland, Sweden; Department of Surgery in Linköping and Department of Biomedical and Clinical Sciences, Linköping University, Linköping, Sweden; Department of Surgery in Linköping and Department of Biomedical and Clinical Sciences, Linköping University, Linköping, Sweden

## Abstract

**Background:**

Pancreatic surgery is rapidly transitioning towards minimally invasive methods. Positive results have been published regarding the safety and efficacy of laparoscopic distal pancreatectomy, but postoperative quality of life after operation remains relatively unexplored. The aim of this study was to assess the long-term quality of life after open *versus* laparoscopic distal pancreatectomy.

**Methods:**

A long-term analysis of quality-of-life data after laparoscopic and open distal pancreatectomy based on the LAPOP trial (a single-centre, superiority, parallel, open-label, RCT in which patients undergoing distal pancreatectomy were randomized 1 : 1 to either the open or laparoscopic approach). Patients received the quality-of-life questionnaires QLQ-C30 and PAN26 before surgery and at 5–6 weeks, 6 months, 12 months, and 24 months after surgery.

**Results:**

Between September 2015 and February 2019, a total of 60 patients were randomized, and 54 patients (26 in the open group and 28 in the laparoscopic group) were included in the quality-of-life analysis. A significant difference was observed in six domains in the mixed model analysis, with better results among patients who underwent laparoscopic surgery. At the 2-year measurement, a statistically significant difference between groups was seen in three domains, and a clinically relevant difference of 10 or more was seen in 16 domains, with better results among the patients who underwent laparoscopic resection.

**Conclusion:**

Considerable differences were shown in postoperative quality of life after laparoscopic compared with open distal pancreatectomy, with better results among the patients who had undergone laparoscopic resection. Of note, some of these differences persisted up to 2 years after surgery. These results strengthen the ongoing transition from open to minimally invasive pancreatic surgery for distal pancreatectomy. Registration number: ISRCTN26912858 (http://www.controlled-trials.com).

## Teaser

This was a long-term analysis of quality-of-life data after laparoscopic and open distal pancreatectomy based on the randomized clinical trial LAPOP. It was shown that the laparoscopic group had significantly better results in six quality-of-life domains over time. At the 2-year measurement, significant differences were shown in 3 domains and clinically relevant differences in 16 domains, with better results in the laparoscopic group.

## Introduction

Pancreatic surgery is continuously developing and has undergone major changes over recent decades with the increasing implementation of laparoscopic and robotic techniques. The shift to minimally invasive surgery has been fastest for distal resections, where international guidelines currently recommend the minimally invasive approach over the open approach for benign and low-grade malignant tumours. Regarding ductal adenocarcinoma, to date, there is a lack of RCTs, but, based on the available evidence, the Miami international guidelines concluded that minimally invasive distal pancreatectomy seemed to be safe and oncologically equivalent to the open technique^1^.

Differences in postoperative quality of life between laparoscopic and open distal pancreatectomy, however, remain a relatively unexplored topic. A Cochrane systematic review from 2016 investigated differences between the open and laparoscopic techniques based on 12 non-randomized studies, none of which included measurements of health-related quality of life. The conclusion was that there was a need for RCTs to be conducted measuring health-related quality of life with at least 2–3 years of follow-up^2^. This has thus far only been accomplished in one study, the LEOPARD trial from the Netherlands, which reported no significant differences between the groups regarding quality-adjusted life years or the quality-of-life subscales in the long-term follow-up, but did report higher cosmetic satisfaction in the minimally invasive group^3^.

The LAPOP trial was a randomized trial performed in Linköping between 2015 and 2019, where patients were randomized 1 : 1 to open or laparoscopic distal pancreatectomy. The primary outcome was postoperative length of stay, which was significantly shorter in the laparoscopic group, as was the main secondary outcome of time to functional recovery^4^.

This study reports long-term quality-of-life results of the LAPOP trial up to 2 years after surgery^4,5^.

## Methods

### Study design

The LAPOP trial was a single-centre, open-label, parallel, superiority RCT in which patients were randomized 1 : 1 to open or laparoscopic distal pancreatectomy and analysed on an intention-to-treat basis^4,5^. Quality-of-life measurements were performed before surgery at baseline, as well as at 5–6 weeks, 6 months, 12 months, and 24 months after surgery. For the quality-of-life analysis, only patients who had undergone resection and had responded to at least one quality-of-life questionnaire were included. The protocol was approved by the ethics board in the South-East Healthcare Region of Sweden with decision number 2015/39-31. The full study protocol can be found in *Appendix S1*.

### Health-related quality of life

Health-related quality of life was measured using the European Organisation for Research and Treatment of Cancer’s (EORTC’s) QLQ-C30 and PAN26 questionnaires^6^. These have been developed for evaluating health-related quality of life among patients with cancer (QLQ-C30) and pancreatic cancer (PAN26). The questions can be converted into domains using a scoring procedure, resulting in a total of seven functional domains, 24 symptom domains, and one global health status^7^. The PAN26 questionnaire has undergone phase III in its development, but has yet to undergo psychometric testing in a large international group of patients to be fully validated. It is therefore advised to test the internal consistency of the compound domains before using them^8^. Regarding the two domains of pain and pancreatic pain, the former refers to pain in general and the latter more specifically to abdominal/back pain.

### Statistical analysis

The statistical analyses were performed using SPSS^®^ Statistics for Windows, version 28.0 (IBM, Armonk, NY, USA). The internal consistency of compound domains from the PAN26 questionnaire was assessed using Cronbach’s α, and those falling below the suggested limit of 0.7 were excluded from further analyses. The different quality-of-life domains were examined over time using a linear mixed model analysis for repeated measurements, first with and then without interaction, without random effects or covariates and with fixed effects for the variable under investigation and the different time points. The values at the 24-month follow-up were also compared between groups using independent sample *t* tests.

In the statistical analyses, a two-tailed *P* value of <0.05 was considered significant. In the quality-of-life measurements, a difference of 10 or more was considered clinically relevant^9^.

Comparative values for the QLQ-C30 domains among the general population were obtained from a large set of norms among the Swedish population published by Michelson *et al*.^10^. From these, a group of age- and sex-matched controls were calculated. As there were no available data in this publication for patients aged 80 years or older, the values for patients aged 70–79 years were also used for patients aged 80 years or older.

### Sensitivity analyses

For the analysis of sensitivity, the mixed model analyses were replicated first excluding patients with pancreatic adenocarcinoma, then patients with a Clavien–Dindo complication score^11^ of IIIa or higher, and lastly patients who had undergone a spleen-preserving procedure. Because of the results of the latter, separate mixed model analyses were done comparing patients who had undergone splenectomy with those who had not.

## Results

### Patients

**Fig. 1:**
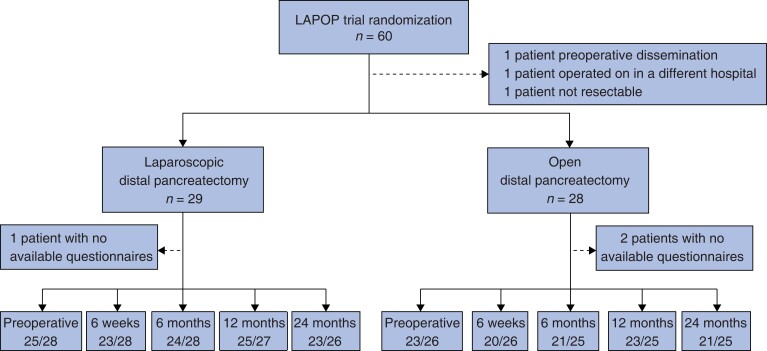
Patients and response rates to the EORTC questionnaires in the two treatment arms The response rates are denoted as the number of patients replying out of the number of patients alive at the time of measurement.

Between September 2015 and February 2019, a total of 60 patients were randomized. Two patients did not undergo resection, one patient underwent surgery at a different hospital, and an additional three patients did not respond to any quality-of-life questionnaires. Quality-of-life questionnaires were sent to the remaining 54 patients, 28 in the laparoscopic group and 26 in the open group (*Fig. 1*).

The patient characteristics as described in the original publication were relatively comparable, with a mean age of 68 years in the laparoscopic group and 63 years in the open group, a male-to-female ratio of 19 : 10 in the laparoscopic group and 15 : 14 in the open group; and a mean BMI of 27 kg/m^2^ in the laparoscopic group and 28 kg/m^2^ in the open group^4^. There was a difference in the proportion of pancreatic adenocarcinoma, with one patient in the open group and six patients in the laparoscopic group being affected. Four patients in the laparoscopic group and six patients in the open group had a Clavien–Dindo complication score of IIIa or higher^12^. Twenty-one patients in the open group and 18 patients in the laparoscopic group underwent splenectomy together with the procedure.

### Internal consistency of PAN26 variables

In Cronbach’s α test, three of the compound variables in the PAN26 questionnaire were below the suggested limit of 0.7: hepatic symptoms, bowel habit, and body image. These domains were therefore excluded from the analyses.

### Quality of life

**Fig. 2:**
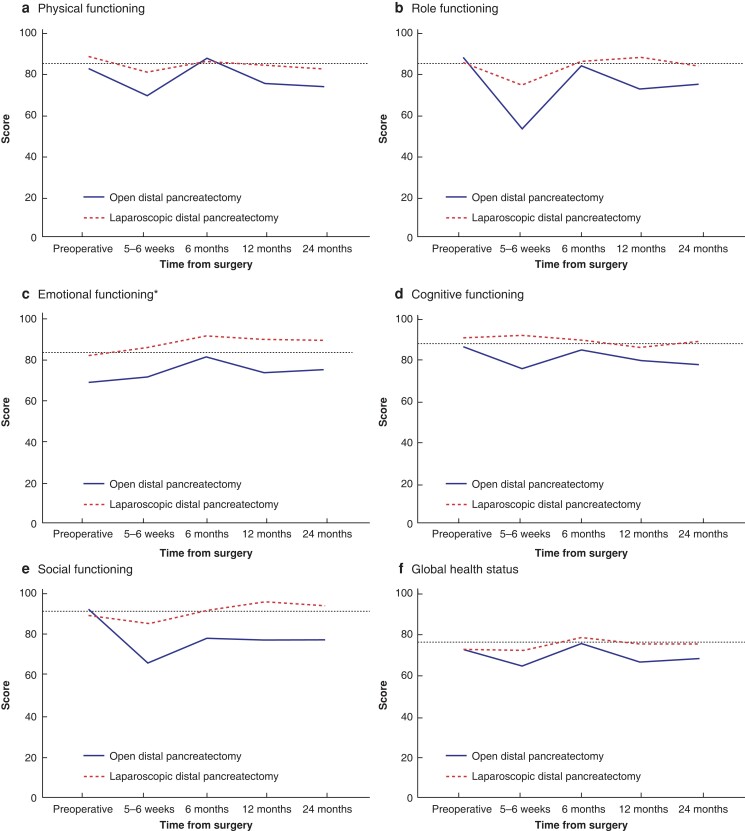
Functional domains and global health status from QLQ-C30 compared between patients who underwent open and laparoscopic distal pancreatectomy **a** Physical functioning. **b** Role functioning. **c** Emotional functioning. **d** Cognitive functioning. **e** Social functioning. **f** Global health status. The *y* axis indicates the scores of the different domains from 0 to 100, where 100 is the best possible. The dotted line shows the reference values among age- and sex-matched controls from the Swedish population. *Statistically significant difference in the mixed model analysis.

**Fig. 3:**
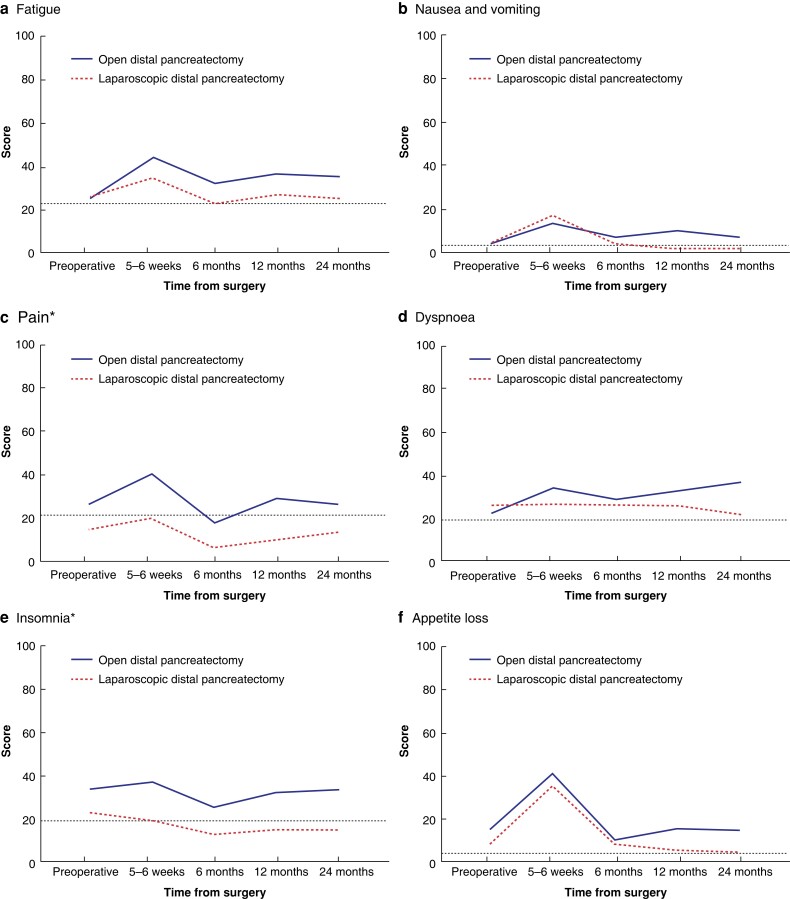

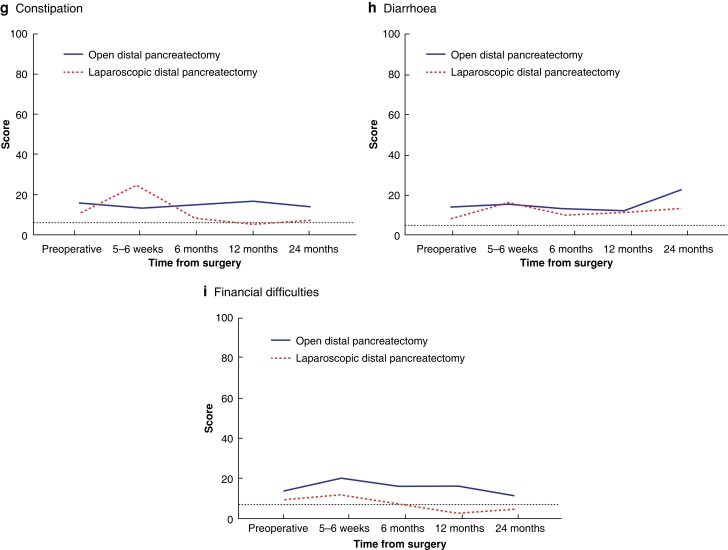
Symptom domains from QLQ-C30 compared between patients who underwent open and laparoscopic distal pancreatectomy **a** Fatigue. **b** Nausea and vomiting. **c** Pain. **d** Dyspnea. **e** Insomnia. **f** Appetite loss. **g** Constipation. **h** Diarrhoea. **i** Financial difficulties. The *y* axis indicates the scores of the different domains from 0 to 100, where 100 is the worst possible (the most symptoms). The dotted line shows the reference values among age- and sex-matched controls from the Swedish population. *Statistically significant difference in the mixed model analysis.

**Fig. 4:**
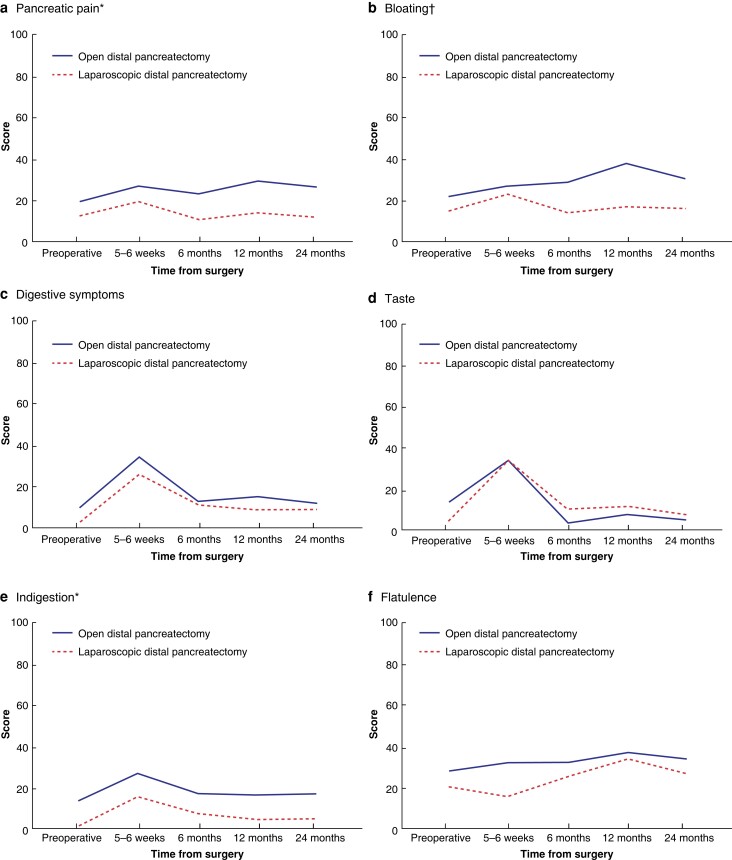

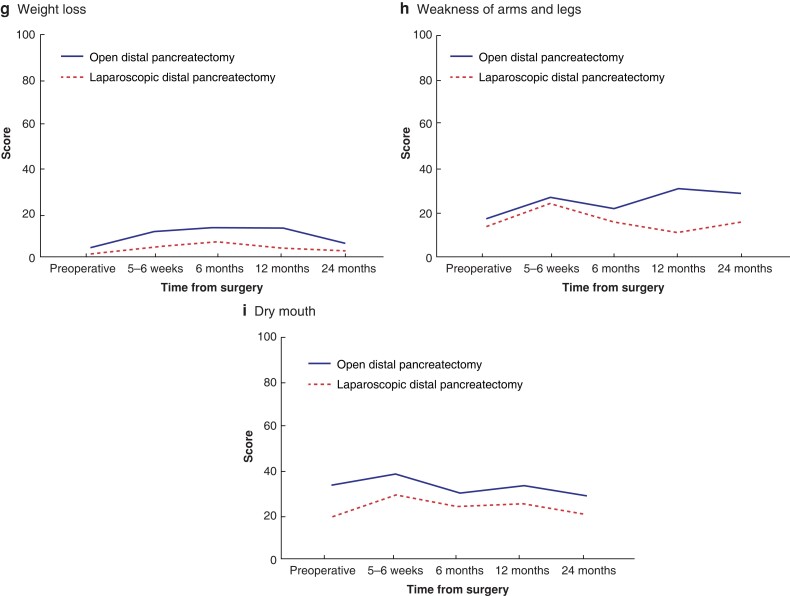
Symptom domains from PAN26 compared between patients who underwent open and laparoscopic distal pancreatectomy **a** Pancreatic pain. **b** Bloating. **c** Digestive symptoms. **d** Taste. **e** Indigestion. **f** Flatulence. **g** Weight loss. **h** Weakness of arms and legs. **i** Dry mouth. The *y* axis indicates the scores of the different domains from 0 to 100, where 100 is the worst possible (the most symptoms). *Statistically significant difference in the mixed model analysis. †Statistically significant difference in the mixed model analysis, but with significant interaction between groups.

**Fig. 5:**
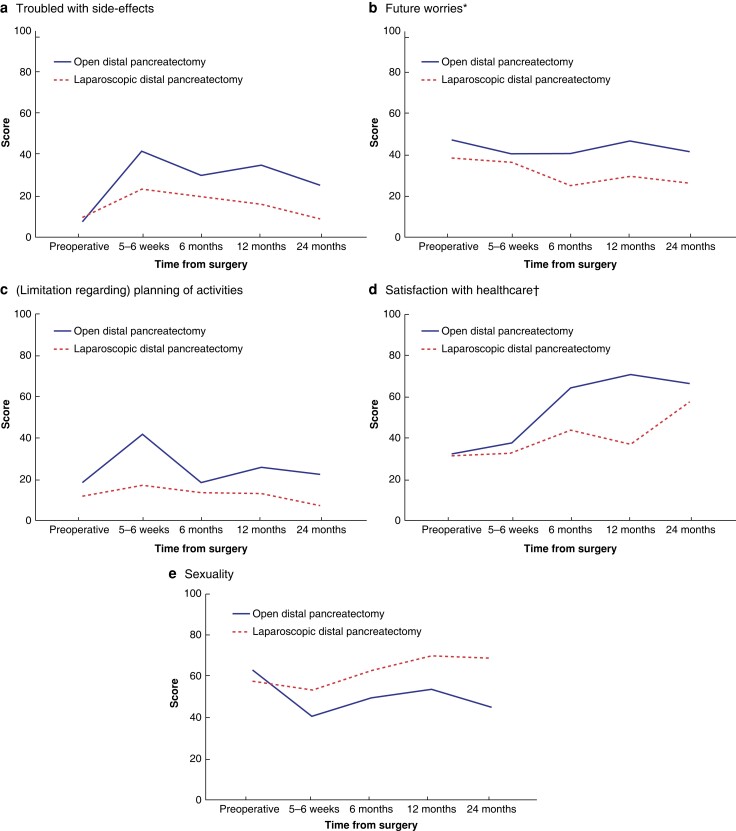
Symptom and functional domains from PAN26 compared between patients who underwent open and laparoscopic distal pancreatectomy **a** Troubled with side-effects. **b** Future worries. **c** (limitation regarding) Planning of activities. **d** Satisfaction with healthcare. **e** Sexuality. The *y* axis indicates the scores of the different domains from 0 to 100, where 100 is the worst possible in domains **a** to **c** (the most symptoms), and the best possible in domains **d** and **e**. *Statistically significant difference in the mixed model analysis. †Statistically significant difference in the mixed model analysis, but with significant interaction between groups.

The development of quality of life over time in both groups is shown in *Figs 2–5*. In the mixed model analysis, significant differences were shown between groups for the domains of emotional functioning, pain, insomnia, pancreatic pain, future worries, and indigestion, with better results among the minimally invasive group. Bloating and satisfaction with healthcare were also significant, but with a significant interaction between the groups. The only variable in favour of open resection was satisfaction with healthcare.

**Fig. 6:**
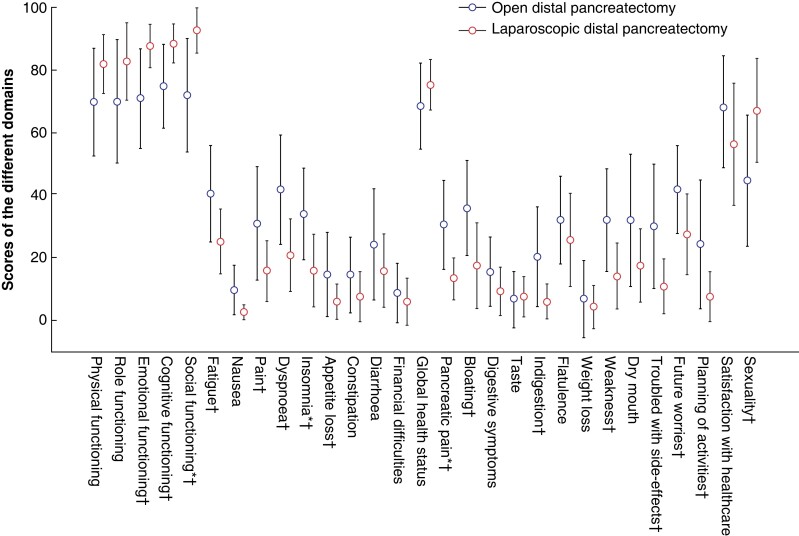
Values at the 24-month follow-up compared between the two treatment arms are shown as the mean with 95 per cent confidence intervals The *y* axis indicates the scores of the different domains from 0 to 100, where 100 is the best possible for all functional domains (physical, role, emotional, cognitive, and social functioning, as well as global health status, satisfaction with healthcare, and sexuality), and the worst possible for all symptom domains (all others). *Statistically significant difference according to *t* test. †Clinically relevant difference of 10 or more.

When comparing only the values from 2 years after surgery, a clinically relevant difference of 10 or more between the groups was seen for the domains of emotional functioning, cognitive functioning, social functioning, fatigue, pain, dyspnoea, insomnia, appetite loss, pancreatic pain, bloating, indigestion, weakness of arms and legs, troubled with side-effects, future worries, (limitation regarding) planning of activities, and sexuality, with better results in the minimally invasive group. The domains of social functioning, insomnia, and pancreatic pain were significantly worse in the open group according to *t* tests (*Fig. 6*).

In the sensitivity analyses with mixed models among the patients without pancreatic ductal adenocarcinoma, the results were unchanged except that the domains of pancreatic pain, bloating, and satisfaction with healthcare were no longer significant. In the analyses with mixed models among the patients with Clavien–Dindo scores of II or less, the results were unchanged except that the domains of insomnia and future worries were no longer significant, and financial difficulties were significantly higher in the open group.

When comparing the groups solely among patients who had undergone splenectomy, only emotional functioning and indigestion remained significantly better in the laparoscopic group. For this reason, separate mixed models analyses were conducted between patients who had undergone splenectomy and patients who had not. Only the domains of dyspnoea and troubled with side-effects significantly differed between the groups, with better results among patients who had undergone spleen-preserving procedures.

*Tables S1 and S2* outline the values of QLQ-C30 and PAN26 for the whole cohort over time and the percentage of missing values for the individual domains.

## Discussion

In this long-term follow-up of quality of life after distal pancreatectomy from a RCT, considerable differences were shown between patients who underwent open and laparoscopic distal pancreatectomy, with a preference for the minimally invasive method. Moreover, many of the domains showed a statistically significant and/or clinically relevant difference as late as 2 years after surgery. With the lack of previous research in this area, this study adds valuable information to strengthen the ongoing worldwide transition from open to minimally invasive distal pancreatectomy. Interestingly, when compared with sex- and age-matched Swedish reference values, the laparoscopic treatment arm approximated the general population values in many of the domains after 12–24 months.

There was a difference between the two groups regarding the proportion of ductal adenocarcinoma. This was countered with a sensitivity analysis excluding patients with this diagnosis, which did not have a major impact on the results. One reason for this could be that the number of ductal adenocarcinomas was relatively small, affecting only seven of 54 patients. Interestingly, a higher proportion of ductal adenocarcinomas was seen in the group with better quality of life.

The number of significant domains dropped to only two when comparing only patients who had undergone splenectomy between treatment arms. However, when doing separate analyses comparing all patients who had undergone a spleen-preserving procedure with those who had not, not many differences were shown. Therefore, the difference in the sensitivity analysis is interpreted as resulting from a lack of power when excluding 15 patients from a sample of only 54. Although there is a lack of studies on the effect of splenectomy together with distal pancreatectomy on quality of life, one small study published in 2016 comparing conventional laparoscopic distal pancreatectomy with the spleen-preserving alternative found no significant differences in overall quality of life for any of the measuring points^13^. The effect of splenectomy together with distal pancreatectomy on quality of life remains an interesting and important question for future studies.

When compared with the only available RCT on this subject, the LEOPARD^3^ study, this study showed more pronounced differences between groups, with a preference for the laparoscopic technique. With a relatively similar approach and baseline characteristics, there is no obvious explanation for this difference. However, it should be noted that both studies are relatively small, with 60 patients originally randomized in our study and 108 patients originally randomized in the LEOPARD study. When the results and follow-up quality-of-life measurements from the ongoing international, multicentre DIPLOMA trial^14^ are ready, the evidence regarding the impact of minimally invasive *versus* open distal pancreatic resection will be considerably strengthened.

As minimally invasive surgery moves towards robot-assisted methods, there is a need to evaluate how this transition affects patients’ health-related quality of life. In a recent study using propensity score matching to evaluate differences between robotic and laparoscopic distal pancreatectomy, De Pastena *et al*.^15^ concluded that the robotic approach seemed to increase quality of life in four of the QLQ-C30 and PAN26 domains. This remains an area with a great need for further investigation.

The use of patient-reported outcome measures such as the QLQ-C30 and PAN26 questionnaires in pancreatic research has increased considerably over recent decades and is likely to continue expanding in importance in the future as treatments improve and focus moves from traditional clinical measurements to the physical and emotional well-being of patients^16^. Apart from the use in research, the implementation of patient-reported outcome measures in clinical practice has been shown to improve patient satisfaction and patient–provider communication, as well as to increase detection of unrecognized problems^17^.

This study has some limitations. First, it is based on the results of the QLQ-C30 and PAN26 questionnaires that were developed to evaluate symptoms among cancer and pancreatic cancer patients, respectively, and there were also patients with benign diagnoses in this study. However, these questionnaires have been widely used in the field of pancreatic surgery to evaluate postoperative quality of life^18–21^ and have also been reported to be appropriate in the measurement of quality of life among patients with chronic pancreatitis^22^. Another limitation in terms of the use of population-based reference values was that no reference values were found for the PAN26 domains or for the QLQ-C30 domains for patients aged 80 years or older. As such patients only represented a fraction of the patients, it was not believed to have any significant impact on the data. Third, the study had a small sample size, which must be taken into account when interpreting the results, and which also made it underpowered to examine subgroups of patients such as those who had undergone splenectomy together with the procedure.

Some strengths of the study were the high response rates to the questionnaires up to 2 years after surgery, and the fact that it was based on data from a RCT.

## Supplementary Material

zrad002_Supplementary_Data

## Data Availability

The data used in this research will be made available upon request to the corresponding author.
