# Supplementary material for: Quality of life after open versus laparoscopic distal pancreatectomy: long-term results from a randomized clinical trial
Source: BJS Open. 2023 Mar 9;7(2):zrad002. doi: 10.1093/bjsopen/zrad002 (PMC9997774; doi:10.1093/bjsopen/zrad002)
Supplement: zrad002_Supplementary_Data [file zrad002_supplementary_data.docx]

**Quality of life after open versus laparoscopic distal pancreatectomy: Long-term results from a randomized clinical trial**

Karin Johansen^1^, Anna Lindhoff Larsson^1^, Linda Lundgren^1^, Thomas Gasslander^1^, Claes Hjalmarsson^2^, Per Sandström^1^, Bergthor Björnsson^1^

^1^ Department of Surgery in Linköping and Department of Biomedical and Clinical Sciences, Linköping University, Linköping, Sweden

^2^ Department of Surgery, Hospital of Halland, Halland

Corresponding author in the review process: Karin Johansen, phone +354 7754318, Email: karin.johansen@liu.se

Official corresponding author and requests for reprints: Bergthor Björnsson, Department of Surgery, Linköping University Hospital, 58175 Linköping. Phone: +46 10 10 33666. Email: bergthor.bjornsson@liu.se

**Supplementary Materials – Index**

| **Supplementary Figures and Tables** |  |
| --- | --- |
| Table S1: Development of EORTC QLQ-C30 domains over time | *page 2* |
| Table S2: Development of EORTC PAN26 domains over time. | *page 3* |
| **Supplementary Appendixes**  Appendix S1: Original study protocol | *page 4* |
|  |  |

**Supplementary Figures and Tables**

Table S1. Development of EORTC QLQ-C30 domains over time.

| Domain (mean ± SD) | Preoperative | 5-6 weeks | 6 months | 12 months | 24 months | Missing data |
| --- | --- | --- | --- | --- | --- | --- |
| Physical functioning | 85 ± 17 | 75 ± 21 | 86 ± 18 | 79 ± 24 | 78 ±26 | 1% |
| Role functioning | 86 ± 23 | 64 ± 31 | 84 ± 25 | 80 ± 30 | 79 ± 31 | 1% |
| Emotional functioning | 76 ± 23 | 79 ± 23 | 86 ± 19 | 82 ± 22 | 82 ± 24 | <1% |
| Cognitive functioning | 88 ± 18 | 84 ± 23 | 87 ± 18 | 82 ± 20 | 83 ± 20 | <1% |
| Social functioning | 89 ± 16 | 75 ± 32 | 84 ± 22 | 86 ± 24 | 85 ± 27 | <1% |
| Fatigue | 25 ± 21 | 39 ± 26 | 27 ± 21 | 31 ± 24 | 30 ± 26 | 1% |
| Nausea and vomiting | 4 ± 9 | 15 ± 23 | 5 ± 12 | 5 ± 14 | 4 ± 11 | 1% |
| Pain | 20 ± 25 | 29 ± 30 | 11 ± 18 | 19 ± 26 | 19 ± 28 | <1% |
| Dyspnoea | 24 ± 26 | 29 ± 29 | 27 ± 28 | 28 ± 28 | 29 ± 29 | 1% |
| Insomnia | 28 ± 30 | 27 ± 35 | 18 ± 24 | 23 ± 26 | 23 ± 27 | 1% |
| Appetite loss | 11 ± 22 | 37 ± 40 | 9 ± 20 | 10 ± 20 | 9 ± 21 | 1% |
| Constipation | 13 ± 23 | 19 ± 27 | 11 ± 21 | 11 ± 23 | 11 ± 21 | 1% |
| Diarrhoea | 11 ± 21 | 16 ± 22 | 11 ± 26 | 11 ± 21 | 17 ± 29 | <1% |
| Financial difficulties | 11 ± 28 | 16 ± 25 | 11 ± 25 | 9 ± 24 | 8 ± 21 | <1% |
| Global health status | 72 ± 21 | 68 ± 19 | 76 ± 20 | 70 ± 22 | 71 ± 22 | <1% |

Table S2. Development of EORTC PAN26 domains over time.

| Domain (mean ± SD) | Preoperative | 2 months | 6 months | 12 months | 24 months | Missing data |
| --- | --- | --- | --- | --- | --- | --- |
| Pancreatic pain | 16 ± 19 | 23 ± 22 | 16 ± 18 | 21 ± 24 | 19 ± 22 | 1% |
| Bloating | 18 ± 27 | 25 ± 27 | 20 ± 24 | 27 ± 29 | 23 ± 29 | 1% |
| Digestive symptoms | 6 ± 17 | 30 ± 26 | 12 ± 20 | 12 ± 20 | 10 ± 18 | 1% |
| Taste | 8 ± 21 | 33 ± 31 | 7 ± 18 | 9 ± 18 | 6 ± 15 | 1% |
| Indigestion | 7 ± 19 | 20 ± 26 | 11 ± 19 | 10 ± 18 | 10 ± 23 | 3% |
| Flatulence | 24 ± 28 | 23 ± 27 | 28 ± 28 | 35 ± 33 | 30 ± 31 | 1% |
| Weight loss | 3 ± 9 | 8 ± 16 | 10 ± 27 | 9 ± 20 | 5 ± 18 | 1% |
| Weakness of arms and legs | 16 ± 24 | 25 ± 25 | 19 ± 24 | 21 ± 28 | 22 ± 27 | 2% |
| Dry mouth | 26 ± 30 | 33 ± 37 | 27 ± 34 | 29 ± 32 | 24 ± 33 | 1% |
| Troubled with side-effects | 9 ± 20 | 32 ± 29 | 24 ± 29 | 25 ± 30 | 17 ± 30 | 2% |
| Future worries | 43 ± 27 | 38 ± 28 | 32 ± 28 | 38 ± 29 | 33 ± 28 | 2% |
| (Limitation regarding) planning of activities | 15 ± 23 | 29 ± 34 | 16 ± 24 | 19 ± 32 | 14 ± 30 | 1% |
| Satisfaction with health care | 32 ± 34 | 35 ± 35 | 53 ± 38 | 53 ± 36 | 61 ± 39 | 3% |
| Sexuality | 60 ± 38 | 47 ± 37 | 56 ± 40 | 62 ± 37 | 57 ± 40 | 8% |

**Supplementary Appendixes**

Appendix S1: Original study protocol

**Study protocol**

RCT-Laparoscopic vs open distal pancreatectomy in an unselected patient cohort

**Ethical approval:** 2015/39-31

**KCTR registration number:**

**Coordinating investigator:** Bergthór Björnsson

**Department of clinical and experimental medicine, division of Surgery, University Hospital, Linköping Sweden**

**Investigator signature………………………………………………………………………….**

**Date……………….**

**Site Investigator:**

**Addition after EPN approval (kick-off meeting CH and BB 10/9):**

**Information that the patient is participating in the study is to be registered in medical record**

**24 Ch silicon drain is to be inserted in the operation, towards the divided pancreas, behind the stomach**

**Open operations are done through midline incision**

**Table of contents**

**Introduction**

**Hypothesis**

**Target population**

**Study objectives**

**Schedule**

**Study design**

**Eligibility criteria**

**Randomisation**

**Pretreatment evaluation**

**Treatment plan**

**Follow up**

**Quality of life assessment**

**Definitions of outcomes**

**Statistical methodology**

**Quality assurance and monitoring**

**Time plan**

**Publication**

**Ethical aspects**

**Investigators**

**References**

**Introduction**

In the late 80s and early 90s laparoscopy within the field of surgery became increasingly common. The laparoscopic cholecystectomy followed by adrenalectomy and splenectomy as well as appendectomy became standard procedures ([1-6](#_ENREF_1)). Today laparoscopic resections for colon cancer are considered safe and oncologically equivalent to open surgery ([7](#_ENREF_7)). In fact, the introduction of laparoscopy in surgery may be described as the largest uncontrolled unrandomized trial ever undertaken.

Regarding malignancies of the pancreas, the initial use of laparoscopy was in staging and palliative procedures ([8-11](#_ENREF_8)).

In 1994 the possibility of laparoscopic distal pancreatectomy (LDP) was assessed in porchine model and the authors concluded that the procedure should be possible to perform in humans ([12](#_ENREF_12)). In the same year Gagner performed a laparoscopic Whipple in a case of chronic pancreatitis ([13](#_ENREF_13)).

Only two years later, Gagner reported retrospectivly, laparoscopic distal pancreatectomy in eight patients with presumed benign tumours (one unclear) as well as four enucleations, however, this is also the first report on LDP in a patient with adenocarcinoma as one of the tumours was an cystadenocarcinoma ([14](#_ENREF_14)). Cuschieri reported the procedure done in five patients for chronic pancreatitis and in the same year case reports further supporting this new approach was published ([15](#_ENREF_15), [16](#_ENREF_16)). The procedure was in its infancy and was reserved for other indications than adenocarcinoma of the pancreas ([17](#_ENREF_17), [18](#_ENREF_18)).

A year later the first report on laparoscopic distal pancreatectomy from USA was published ([19](#_ENREF_19)). In the same year (1997) the first case report on an issue later to become widely discussed, the preservation of the spleen, was published ([20](#_ENREF_20)).

The second report including adenocarcinoma of the pancreas included 2 LDP, of which one was for adenocarcinoma ([21](#_ENREF_21)).

The spleen preservation was further established in the literature in 1999 ([22](#_ENREF_22), [23](#_ENREF_23)). The higher rate of spleen preservation seen with LPD than Open distal pancreatectomy (OPD) has been considered positive in general while it is known that resections of the splenic vessels adds to the oncological radicality of the operation and may increase the number of lymph nodes retrieved ([24](#_ENREF_24)).

In a series of 15 patient operated on with LDP between 1993 and 2000 two were found to have adenocarcinoma of the pancreas, margin status and lymph node number for these patients is unknown ([25](#_ENREF_25)).

Fernández-Cruz reported on LDP for chronic pancreatitis in 2002 and compared the results to open surgery. In this small material he found that the LDP was feasible for this patient category ([26](#_ENREF_26)).

In a series of 6 attempts (5 successful) LDP from 2002 two of the patients were operated on for adenocarcinoma ([27](#_ENREF_27)). One was operated on with curative intention and was reported alive and free of tumour at 2 year follow up. The other was operated on with palliative intention and a liver metastasis was removed at the same operation. The number of lymph nodes resected was reported as 19 respective 6 and margin status was R0 respective Rx. Although this report does not compare LDP to ODP it is the first to report on those oncological findings.

When the subject of LDP was reviewed in 2003 a total of 47 cases reported in 18 articles was found. Of those 3 were adenocarcinomas and the indication for LDP was still found to be non-malignant lesions ([28](#_ENREF_28)). At the same time the indications were evolving from generally including neuroendocrine pancreatic tumours and pancreatitis to including cystic neoplasias of the pancreas as well ([29](#_ENREF_29), [30](#_ENREF_30)).

Early results from 1997-2003 published in 2004 retrospectively analyse 12 LDP including 4 adenocarcinomas, however lymph node count and margin status is not mentioned ([31](#_ENREF_31)). In another report from 2004 early results on 17 LDPs performed in 1997-2002 are presented . Four had adenocarcinoma (3 successfully resected with LDP) and two had positive surgical margins, lymph node count is not provided

In 2005 a large retrospective European multicentre study summarized the experience of laparoscopic distal pancreatectomy from 1995 to 2002 and found that out of 127 patients,6 had adenocarcinomas of the pancreas ([32](#_ENREF_32)). In the same year a report on 21 prospectively registrated patients undergoing LDP including 2 adenocarcinomas showed the feasibility of the method with low morbidity rate ([33](#_ENREF_33)).

In a retrospective analysis of prospectively sampled data on 16 hand-assisted LDP done in 2002-2004 two were done for suspected or confirmed by fine needle aspiration (n=1) adenocarcinoma. The final pathology showed one case of chronic pancreatitis and one of adenosquamous carcinoma, The R0 frequency was found to be 76% and the mean number of lymph nodes resected was 5.5 ([34](#_ENREF_34)). In another article published in 2006 15 cases of attemted LDP resulted in 3 conversions to ODP and these 3 were the only cases of pancreatic adenocarcinoma in the LDP group, lymp node count a margin status is not described ([35](#_ENREF_35)). In a report on 19 LDPs done for benign or low-grade malignant lesions were 3 adenocarcinomas were found on the final pathology but lymph node count and surgical margins are not mentioned ([36](#_ENREF_36)).

In 2007 a single-centre analysis of 103 LDP between 1998 and 2007 including 13 ductal adenocarcinomas was presented with 90% R0 resections in that subgroup ([37](#_ENREF_37)) In this report suspected and overt malignant lesions were operated on according to the principle of Radical Antigrade Modular PancreatoSplenectomy (RAMPS) principle, 3 of the operations were converted to open surgery all of which included extended en block resection of other organs. In median 14.5 lymph nodes were resected but tumour size was not reported.

In a single institution retrospective analysis of 58 LDP performed during the period of 1999 to 2005 were described. The group includes 5 ductal adenocarcinomas as well as one mucinous cystadenocarcinoma but unfortunately surgical margin status or lymph node harvesting is not mentioned ([38](#_ENREF_38)). The surgical approach was a retrograde dissection of the pancreas.

The first multicentre experience from USA was reported in 2008 (data from 2002 to 2006), in 8 centres (of which 3 were defined as high volume, >30 LDP) 159 LDP were attempted with 20 (13%) converted to open surgery. Of those 16 were shown to have adenocarcinoma ([39](#_ENREF_39)). At the same time reports on single-center experiences with reasonable number of patients became to emerge. In a retrospective case matched material of LDP (n=31) was shown to be equivalent to ODP (n=62) regarding complications, operation time and bleeding but the hospital stay was shorter in the LDP group, there were however no ductal adenocarcinomas in the group ([40](#_ENREF_40)).

An early 10 year report (1996 to 2006) of 46 attempted LDP in 5 institutions includes 9 ductal adenocarcinomas (as well as 1 mucinous cystadenocarcinoma). Twelve patients were converted to open surgery (including 4 ductal adenocarcinomas). Unfortunately surrogate oncological markers are not included in the report ([41](#_ENREF_41)).

In a retrospective analysis of 128 patients operated on for benign lesions of the pancreatic body or tail where the patients got to choose either LDP or ODP 93 patients were operated on with LDP and 35 with ODP ([42](#_ENREF_42)). The results were similar except for hospital stay that was significantly shorter in the LDP group and splenic preservation that was more common in the LDP group.

25 successful LDP (31 attempted ) that were prospectively evaluated were described, one may have had adenocarcinoma although this cannot be confirmed because other laparoscopical pancreatic procedures are described in the article as well. However all patients operated upon with malignant diagnosis had negative surgical margins. Lymph node numbers are not given ([43](#_ENREF_43)).

During the first 12-14 years from the introduction of LDP about 50 cases of adenocarcinoma of the pancreas had emerged among the benign and low-malignant diagnosis that were considered indications for LDP. Unfortunately no randomized study has been performed on the applicability of LDP for adenocarcinoma of the pancreas or for other diseases. Multiple meta-analyses have shown LDP to be superior compared to ODP regarding hospital stay, and perioperative bleeding but the data comes from selected patient cohorts ([44-46](#_ENREF_44)).

In Sweden, open surgery is the most used method in patients with pancreatic tail tumour independent if they are malignant or benign. In major HPB centres globally the opposite is the rule. A randomized study is therefore very important to evaluate this topic as the knowledge today is based on case studies.

**Hypothesis:**

LDP, with or without the use of robot, shortens hospital stay and reduces bleeding compared to ODP in unselected patients undergoing distal pancreatectomy with or without splenectomy.

**Target population**

All patients identified at multidisciplinary conference in need of distal pancreatectomy regardless of tumour type.

**Study objectives:**

**Primary objective**:

To compare length of stay in hospital after laparoscopic- and open distal pancreatectomy.

**Secondary objectives:**

To compare perioperative bleeding during LDP and ODP

To compare postoperative pain after LDP and ODP

To compare the use of analgetics after LDP and ODP

To evaluate quality of life postoperatively after LDP and ODP

To analyze costs associated with LDP and ODP

To compare complications in LDP and ODP

To evaluate survival

Survival will be analysed after 2 years in a clinical manner. Deaths and diagnosed recurrences will be recorded. In cases of suspicion of recurrence a CT scan will be performed.

**Schedule**

**Patient population**

Patients over 18 years of age.

At multidisciplinary conference found the patient to have an indication for distal pancreatectomy (with or without splenectomy)

Suitable for surgery (physiologically operable).

Possible to achieve R0 resection without resection of other organs (besides the spleen)

Planned division of pancreas to the left of the SMV

**Study design**

**6.1** The study is designed as a prospective 1:1 randomised non-blinded trial.

**Eligibility criteria**

**Inclusion criteria**

7.1 Patients with lesion in the body or tail of the pancreas demanding surgery (indication set by multidisciplinary conference).

7.2 Operable patient (as the local preoperatively evaluation dictates).

7.3 Possibility to achieve R0-resection without resection of additional organs (besides the spleen)

Patients with performance status 0-2 according to WHO scale.

Written informed consent.

7.6 Age > 18 years

**Exclusion criteria**

Pregnancy and/or lactation.

Patients being unable to comply with the protocol for reasons of language or cognitive function.

Preoperatively defined need to resect other organs than pancreas and spleen.

Preoperatively defined division line of pancreas to the right of the SMV

**Randomisation**

**8.1** Randomisation will be performed with computer-generated random numbers in block of 10 (5:5). Studygroup allocation will depend upon the contents of envelopes generated in this manner, opened at patient inclusion.

**Pretreatment evaluation**

The following investigations are required prior to protocol entry.

**9.1** CT of the thorax and upper abdomen to assess tumour status performed within 6 weeks of study inclusion.

**9.2** Laboratory studies: Full blood cell count with differential and platelet counts, creatinine, liver function tests, performed within 1 month of study inclusion. PK-INR

**Treatment plan**

**10.1** Patients can be included if they have visible tumour in the body or tail of the pancreas without extension to other organs (excluding the spleen).

**10.2** The treatment plan must be proposed by a multidisciplinary team (MDT) conference, which also identifies the patients as suitable for study inclusion.

**10.3 Surgical treatment**

***Preoperative investigations***

Preoperative evaluation of operability is performed according to the protocol above regarding functional status. The inclusion criteria preclude that all patients are resectable.

**Follow-up**

**11.1 Follow-up assessment**

All patients come for an outpatient visit 4-6 weeks after surgery. QOL assessment is done at this visit. Further follow up is outside this study and will be dictated by the pathological diagnosis according to clinical need.

**Quality of life assessment**

**12.1 Quality of life**

Specific attention is to be directed towards quality of life. To this end, simple quality of life assessment questionnaires using a self-assessment linear analogue scale to measure physical well-being, mood, pain, nausea and vomiting, appetite, swallowing and tiredness, will be administered once every 3 months and at the time of the first relapse (see Appendix 3). This will be completed with EORTC QLQ-C30 to which is added the PAN26 module and EQ-5D.

The questionnaires will be filled out by the study subjects att inclusion, after 5-6 week after surgery, at 6 and 12 months postoperatively and 24 months postoperatively.

**Definitions of outcomes**

**13.1 Length of stay:** Number of days spent in the hospital after surgery.

**13.2** Medical length of stay: Defined as no need for intravenous drug administration or fluids as well ambulatory patient able to perform ADL. This does not exclude discharge with drains or urinary catheters

**13.3** **Perioperative bleeding:** The amount of blood lost (ml) during surgery as estimated by nurse (anesthesia) in the operation room. Volume in suction device + weight of cloths – fluids used during operation is used to estimate blood loss.

**Pain:** Patient perception of pain measured with a Visual Analouge Scale.

**Pain relief:** The number of paracetamol, NSAID and opioid pills given as well as the amount of i.v. and s.c. administrated opioids. All patients are offered PCA (patient controlled analgesia) initially after operation. Epidural anesthesia can be offered if deemed necessary. In those cases this is registered separately.

**Complications:** Registered as the highest Clavien score in each patient. In addition POPF grade B and C will be analysed separately

**Quality of Life:** Quality of life will be measured prior to surgery and 4-6 weeks after surgery. The EORTC QLQ-C30, EORTC PAN26 as well as EQ-5D will be used.

**Cost:** Hospital cost, days (according to schablon), actual OR cost as well as cost for readmission, sick leave etc.

**Lymph nodes resected:** The total number of lymph nodes reported in the pathology report

**R0:** Tumour reported not to extend to the surgical margin in the pathology report

**Statistical methodology**

**14.1 Sample size**

The sample size considerations are based on the primary endpoint hospital stay in an intention to treat manner. One sided power calculation is used as none of the previous publications indicates inferiority for LDP. Assumed mean hospital stay is 5 and 7.5 days for laparoscopic respective open operations. The standard deviation is 3,5 and with type I error = 0.05 and a 0.8 power 25 patients are needed in each group.

The sample size is calculated based on a expected conversion rate of 20%

Due to possible drop-outs 30 patients will be inculded in each group.

**14.2 Analysis plan**

The analyses will be based on the intention to treat principle. Demography, treatment and clinical data will be reported.

Quality of life as measured by the EORTC QLQ-C30, EORTC PAN26 and EQ-5D will be analysed.

Cost-benefit analysis will be performed with regards to the operation, postoperative complications and interventions, days in hospital, need for postoperative outpatient treatment, readmissions for complications, tumor recurrence. Cost for sick leave will be included.

Survival will be analysed in a intention to treat manner with over all survival and DFS (disease free survival) at 2 year follow up. Deaths and diagnosed recurrences will be recorded. In cases of suspicion of recurrence a CT scan will be performed.

**14.3 Adverse events**

Complications to surgical interventions are to be expected in the study and will be registered and classified according to the Clavien-Dindo classification. POPF grade B and C will be separately registered and analysed.

**Quality assurance**

Quality assurance will be done according to GCP rules:

a. Treatment regimens will be recorded (type of surgery).

b. Follow-up, complications.

c. Data reporting and management.
Paper Case Record Forms (CRFs) will be filled on a continuous basis.

d. All operative procedures and related postoperative courses are concomitantly recorded in the Swedish Quality Assurance Register for pancreas, Pankreasregistret.

**Time plan**

First patient will be enrolled in Q1 2015 and 1-2 will be enrolled each month. Based on these calculations the study will be closed in 2018.

**Publication**

**Planned publications**

Study comparing short term outcome, length of stay and peroperative complications

QoL analysis during the first year postoperatively

Cost-benefit analysis of the 2 treatment arms

Survival in the 2 treatment arms

Authors of the publication are those who actively participate in processing of the study protocol, recruiting patients, compiling the results and putting together the article.

**Ethical aspects**

The investigator will ensure that this study is conducted in full conformance with principles of the”Declaration of Helsinki” (including the amendments from Tokyo, Venice and Hong-Kong).

An ethical application will be submitted.

As both open and laparoscopic pancreatic tail resection are established safe surgical methods we find no need for at safety monitor group.

**Investigators**

***Coordinating investigator:***

Bergthór Björnsson, Dep Surgery, Universitetssjukhuset, 58185 Linköping, Sweden

***Investigators at participating sites:***

| Claes Hjalmarsson | Kirurgi | Kalmar |  |  | claes.hjalmarsson@ltkalmar.se |
| --- | --- | --- | --- | --- | --- |
| Linda Lundgren | Kirurgi | Jönköping |  |  | linda.lundgren@ljr.se |
|  |  |  |  |  |  |
|  |  |  |  |  |  |
|  |  |  |  |  |  |

**Reference**

1. Gagner M, Lacroix A, Bolte E. Laparoscopic adrenalectomy in Cushing's syndrome and pheochromocytoma. N Engl J Med. 1992;327(14):1033.

2. Hashizume M, Sugimachi K, Ueno K. Laparoscopic splenectomy with an ultrasonic dissector. N Engl J Med. 1992;327(6):438.

3. Gagner M, Pomp A, Heniford BT, Pharand D, Lacroix A. Laparoscopic adrenalectomy: lessons learned from 100 consecutive procedures. Ann Surg. 1997;226(3):238-46; discussion 46-7.

4. Jacobs JK, Goldstein RE, Geer RJ. Laparoscopic adrenalectomy. A new standard of care. Ann Surg. 1997;225(5):495-501; discussion -2.

5. Katkhouda N, Hurwitz MB, Rivera RT, Chandra M, Waldrep DJ, Gugenheim J, et al. Laparoscopic splenectomy: outcome and efficacy in 103 consecutive patients. Ann Surg. 1998;228(4):568-78.

6. Rescorla FJ, West KW, Engum SA, Grosfeld JL. Laparoscopic splenic procedures in children: experience in 231 children. Ann Surg. 2007;246(4):683-7; discussion 7-8.

7. Buunen M, Veldkamp R, Hop WC, Kuhry E, Jeekel J, Haglind E, et al. Survival after laparoscopic surgery versus open surgery for colon cancer: long-term outcome of a randomised clinical trial. Lancet Oncol. 2009;10(1):44-52.

8. Cuschieri A, Hall AW, Clark J. Value of laparoscopy in the diagnosis and management of pancreatic carcinoma. Gut. 1978;19(7):672-7.

9. Fletcher DR, Jones RM. Laparoscopic cholecystjejunostomy as palliation for obstructive jaundice in inoperable carcinoma of pancreas. Surg Endosc. 1992;6(3):147-9.

10. Nathanson LK. Laparoscopy and pancreatic cancer: biopsy, staging and bypass. Baillieres Clin Gastroenterol. 1993;7(4):941-60.

11. Nathanson LK. Laparoscopic cholecyst-jejunostomy and gastroenterostomy for malignant disease. Surg Oncol. 1993;2 Suppl 1:19-24.

12. Soper NJ, Brunt LM, Dunnegan DL, Meininger TA. Laparoscopic distal pancreatectomy in the porcine model. Surg Endosc. 1994;8(1):57-60; discussion -1.

13. Gagner M, Pomp A. Laparoscopic pylorus-preserving pancreatoduodenectomy. Surg Endosc. 1994;8(5):408-10.

14. Gagner M, Pomp A, Herrera MF. Early experience with laparoscopic resections of islet cell tumors. Surgery. 1996;120(6):1051-4.

15. Cuschieri A, Jakimowicz JJ, van Spreeuwel J. Laparoscopic distal 70% pancreatectomy and splenectomy for chronic pancreatitis. Ann Surg. 1996;223(3):280-5.

16. Sussman LA, Christie R, Whittle DE. Laparoscopic excision of distal pancreas including insulinoma. Aust N Z J Surg. 1996;66(6):414-6.

17. Cuschieri A. Laparoscopic Pancreatic Resections. Semin Laparosc Surg. 1996;3(1):15-20.

18. Salky BA, Edye M. Laparoscopic pancreatectomy. Surg Clin North Am. 1996;76(3):539-45.

19. Clark GJ, Onders RP, Knudson JD. Laparoscopic distal pancreatectomy procedures in a rural hospital. Aorn J. 1997;65(2):334, 7-43.

20. Tihanyi TF, Morvay K, Nehez L, Winternitz T, Rusz Z, Flautner LE. Laparoscopic distal resection of the pancreas with the preservation of the spleen. Acta Chir Hung. 1997;36(1-4):359-61.

21. Santoro E, Carlini M, Carboni F. Laparoscopic pancreatic surgery: indications, techniques and preliminary results. Hepatogastroenterology. 1999;46(26):1174-80.

22. Ueno T, Oka M, Nishihara K, Yamamoto K, Nakamura M, Yahara N, et al. Laparoscopic distal pancreatectomy with preservation of the spleen. Surg Laparosc Endosc Percutan Tech. 1999;9(4):290-3.

23. Vezakis A, Davides D, Larvin M, McMahon MJ. Laparoscopic surgery combined with preservation of the spleen for distal pancreatic tumors. Surg Endosc. 1999;13(1):26-9.

24. Andren-Sandberg A, Wagner M, Tihanyi T, Lofgren P, Friess H. Technical aspects of left-sided pancreatic resection for cancer. Dig Surg. 1999;16(4):305-12.

25. Patterson EJ, Gagner M, Salky B, Inabnet WB, Brower S, Edye M, et al. Laparoscopic pancreatic resection: single-institution experience of 19 patients. J Am Coll Surg. 2001;193(3):281-7.

26. Fernandez-Cruz L, Saenz A, Astudillo E, Pantoja JP, Uzcategui E, Navarro S. Laparoscopic pancreatic surgery in patients with chronic pancreatitis. Surg Endosc. 2002;16(6):996-1003.

27. Barlehner E, Anders S, Schwetling R. Laparoscopic resection of the left pancreas: technique and indication. Dig Surg. 2002;19(6):507-10.

28. Tagaya N, Kasama K, Suzuki N, Taketsuka S, Horie K, Furihata M, et al. Laparoscopic resection of the pancreas and review of the literature. Surg Endosc. 2003;17(2):201-6.

29. Ammori BJ. Pancreatic surgery in the laparoscopic era. Jop. 2003;4(6):187-92.

30. Fernandez-Cruz L, Martinez I, Gilabert R, Cesar-Borges G, Astudillo E, Navarro S. Laparoscopic distal pancreatectomy combined with preservation of the spleen for cystic neoplasms of the pancreas. J Gastrointest Surg. 2004;8(4):493-501.

31. Lebedyev A, Zmora O, Kuriansky J, Rosin D, Khaikin M, Shabtai M, et al. Laparoscopic distal pancreatectomy. Surg Endosc. 2004;18(10):1427-30.

32. Mabrut JY, Fernandez-Cruz L, Azagra JS, Bassi C, Delvaux G, Weerts J, et al. Laparoscopic pancreatic resection: results of a multicenter European study of 127 patients. Surgery. 2005;137(6):597-605.

33. Dulucq JL, Wintringer P, Stabilini C, Feryn T, Perissat J, Mahajna A. Are major laparoscopic pancreatic resections worthwhile? A prospective study of 32 patients in a single institution. Surg Endosc. 2005;19(8):1028-34.

34. D'Angelica M, Are C, Jarnagin W, DeGregoris G, Coit D, Jaques D, et al. Initial experience with hand-assisted laparoscopic distal pancreatectomy. Surg Endosc. 2006;20(1):142-8.

35. Velanovich V. Case-control comparison of laparoscopic versus open distal pancreatectomy. J Gastrointest Surg. 2006;10(1):95-8.

36. Corcione F, Marzano E, Cuccurullo D, Caracino V, Pirozzi F, Settembre A. Distal pancreas surgery: outcome for 19 cases managed with a laparoscopic approach. Surg Endosc. 2006;20(11):1729-32.

37. Fernandez-Cruz L, Cosa R, Blanco L, Levi S, Lopez-Boado MA, Navarro S. Curative laparoscopic resection for pancreatic neoplasms: a critical analysis from a single institution. J Gastrointest Surg. 2007;11(12):1607-21; discussion 21-2.

38. Melotti G, Butturini G, Piccoli M, Casetti L, Bassi C, Mullineris B, et al. Laparoscopic distal pancreatectomy: results on a consecutive series of 58 patients. Ann Surg. 2007;246(1):77-82.

39. Kooby DA, Gillespie T, Bentrem D, Nakeeb A, Schmidt MC, Merchant NB, et al. Left-sided pancreatectomy: a multicenter comparison of laparoscopic and open approaches. Ann Surg. 2008;248(3):438-46.

40. Eom BW, Jang JY, Lee SE, Han HS, Yoon YS, Kim SW. Clinical outcomes compared between laparoscopic and open distal pancreatectomy. Surg Endosc. 2008;22(5):1334-8.

41. Taylor C, O'Rourke N, Nathanson L, Martin I, Hopkins G, Layani L, et al. Laparoscopic distal pancreatectomy: the Brisbane experience of forty-six cases. HPB (Oxford). 2008;10(1):38-42.

42. Kim SC, Park KT, Hwang JW, Shin HC, Lee SS, Seo DW, et al. Comparative analysis of clinical outcomes for laparoscopic distal pancreatic resection and open distal pancreatic resection at a single institution. Surg Endosc. 2008;22(10):2261-8.

43. Sa Cunha A, Rault A, Beau C, Laurent C, Collet D, Masson B. A single-institution prospective study of laparoscopic pancreatic resection. Arch Surg. 2008;143(3):289-95; discussion 95.

44. Jin T, Altaf K, Xiong JJ, Huang W, Javed MA, Mai G, et al. A systematic review and meta-analysis of studies comparing laparoscopic and open distal pancreatectomy. HPB (Oxford). 2012;14(11):711-24.

45. Nigri GR, Rosman AS, Petrucciani N, Fancellu A, Pisano M, Zorcolo L, et al. Metaanalysis of trials comparing minimally invasive and open distal pancreatectomies. Surg Endosc. 2011;25(5):1642-51.

46. Sui CJ, Li B, Yang JM, Wang SJ, Zhou YM. Laparoscopic versus open distal pancreatectomy: a meta-analysis. Asian J Surg. 2012;35(1):1-8.
